# Cross-Instrument Data Utilization Based on Laser-Induced Breakdown Spectroscopy (LIBS) for the Identification of *Akebia* Species

**DOI:** 10.3390/bioengineering12090964

**Published:** 2025-09-08

**Authors:** Yuge Liu, Qianqian Wang, Tianzhong Luo, Zhifang Zhao, Leifu Wang, Shuai Xu, Hao Zhou, Jiquan Zhao, Zixiao Zhou, Geer Teng

**Affiliations:** 1 School of Optics and Photonics, Beijing Institute of Technology, Beijing 100081, China; 3220230643@bit.edu.cn (Y.L.); qqwang@bit.edu.cn (Q.W.);; 2 Key Laboratory of Photonic Information Technology, Ministry of Industry and Information Technology, Beijing Institute of Technology, Beijing 100081, China; 3 National Key Laboratory on Near-Surface Detection, Beijing 100072, China; 4 Yangtze Delta Region Academy of Beijing Institute of Technology, Jiaxing 314033, China; 5 Institute of Biomedical Engineering, Department of Engineering Science, University of Oxford, Oxford OX3 7LD, UK

**Keywords:** laser-induced breakdown spectroscopy, traditional Chinese medicine, spectral correction, analysis of variance, density-based spatial clustering of applications with noise

## Abstract

New technologies and equipment for medicine analysis and diagnostics have always been critical in clinical medication and pharmaceutical production. Especially in the field of traditional Chinese medicine (TCM) where the chemical composition is not fully clear, cross-device analysis and identification using the same technology can sometimes even lead to misjudgments. Akebia species, capable of inducing heat clearing, diuresis, and anti-inflammatory effects, show great potential in clinical applications. However, the three commonly used species differ in pharmacological effects and therefore should not be used interchangeably. We proposed a method combining LIBS with random forest for species identification and established a modeling and verification scheme across device platforms. Spectra of three Akebia species were collected using two LIBS systems equipped with spectrometers of different resolutions. The data acquired from the low-resolution spectrometer were used for model training, while the data from the high-resolution spectrometers were used for testing. A spectral correction and feature selection (SCFS) method was proposed, in which spectral data were first corrected using a standard lamp, followed by feature selection via analysis of variance (ANOVA) to determine the optimal number of discriminative features. The highest classification accuracy of 80.61% was achieved when 28 features were used. Finally, a post-processing (PP) strategy was applied, where abnormal spectra in the test set were removed using density-based spatial clustering of applications with noise (DBSCAN), resulting in a final classification accuracy of 85.50%. These results demonstrate that the proposed “SCFS-PP” framework effectively enhances the reliability of cross-instrument data utilization and expands the applicability of LIBS in the field of TCM.

## 1. Introduction

*Akebia* species are a group of vine plants belonging to Ranunculaceae, whose vines and stems are widely used in TCM [1]. The commonly used *Akebia* species medicines mainly include Mutong (*Akebiae Caulis*), Chuan-mutong (*Clematidis Armandii Caulis*), and Guan-mutong (*Aristolochiae Manshuriensis Caulis*). Mutong and Chuan-mutong can be eaten by grinding them to powder or used as decoction pieces for medicinal purposes [2], but their effectiveness is different. Guan-mutong contains aristolochic acids, which possess nephrotoxic and carcinogenic properties [3]. For to this reason, it was officially prohibited by the National Medical Products Administration of China in 2003 [4]. However, due to the similarity in their appearance, these three *Akebia* species are frequently confused and misused [5]. Historically, serious adverse drug events have occurred repeatedly due to the misapplication of Guan-mutong [6]. Due to differences in efficacy and safety, these three *Akebia* species should not be used interchangeably and must be accurately identified.

The general methods to identify TCM are based on direct morphology observation, microscopic identification, thin-layer chromatography (TLC), and high-performance liquid chromatography (HPLC) [7,8]. Among them, direct morphology observation and microscopic identification are simple to perform but easy to be influenced by subjective factors such as operator experience and visual acuity. In contrast, techniques like TLC and HPLC offer high accuracy but typically require complex sample preparation, making them more suitable for research settings rather than production and distribution applications [9,10]. To tackle the challenges in identifying *Akebia* species, recent studies have sought to overcome the limitations of conventional techniques. Yoon et al. developed a classification model based on two-dimensional Fourier transform infrared spectroscopy (2D-FTIR) to distinguish five types of natural herbs containing Mutong [11]. Xu et al. employed droplet digital polymerase chain reaction (PCR) to detect adulteration in Mutong samples [12]. Although these emerging methods show clear accuracy advantages, they still require complex sample preparation steps, such as drying and grinding the samples, as well as multiple buffer washes to eliminate polysaccharide interference. Therefore, rapid and in situ identification technology without complex sample processing remains an important requirement in this field.

As a novel elemental analysis technology, laser-induced breakdown spectroscopy (LIBS) has several advantages, including fast analytical speed, being in situ, and high efficiency [13], which has been applied in deep space exploration [14,15], geological analysis [16], metallurgical analysis [17], biomedical science [18,19], environmental monitoring [20] and so on. In the field of TCM, LIBS technology is commonly combined with machine learning to conduct medicinal material classification, origin identification, and elemental quantification. Zhang et al. combined LIBS with principal component analysis (PCA), linear discriminant analysis (LDA), and support vector machine (SVM) to identify the origin of ginkgo leaves from eight different regions [21]. Huang et al. integrated LIBS with deep learning and proposed a DMC-LIBSAS model to trace the geographical origin of the TCM *Angelica dahurica* [22]. Shen et al. carried out a highly sensitive determination of multiple nutrient elements in *Panax notoginseng* based on LIBS technology and chemometric methods [23]. Zhu et al. used LIBS technology to quantitatively determine the content of harmful elements in licorice [24]. Wei et al. used LIBS combined with least absolute shrinkage and selection operator (LASSO), partial least squares regression (PLSR), and support vector regression (SVR) to detect the adulteration of *Fritillaria thunbergii* in *Fritillaria cirrhosa* [25]. Fang et al. proposed a method for analyzing LIBS data from damaged medicinal leaves, enabling the quantitative determination of cadmium and lead [26]. Chen et al. employed a crater-spectral feature fusion approach to analyze LIBS emission spectra for the rapid detection of cadmium in *Astragalus membranaceus* [27]. Kabir et al. utilized LIBS emission spectroscopy in combination with variable selection and chemometric techniques to detect heavy metals in *Fritillaria cirrhosa* [28].

Although LIBS technology combined with machine learning has been widely applied for the rapid detection of TCM, in most existing studies, the data used for model building and validation are acquired from the same LIBS measurement setup. When data sources are different, researchers often use transfer learning to address this issue. Cui et al. proposed a new approach that integrates transfer learning to jointly utilize in situ Mars spectra and laboratory spectra. A CNN model was first trained on laboratory spectra before refining the model parameters with in situ Mars spectra to achieve higher accuracy [29]. Sun et al. introduced transfer learning into LIBS spectral data processing to effectively mitigate the physical matrix effect in the case of rock analysis for Mars exploration, thereby significantly enhancing the total alkali-silica (TAS) classification accuracy of pellet-based models [30]. Dong et al. developed a dual-mode optical LIBS system combined with spectral correction and feature transfer learning, enabling the accurate online quantitative analysis of coal particle flow in terms of calorific value, volatile matter, and ash content [31]. Vrábel et al. employed a composed model consisting of a variational autoencoder (VAE) and a multilayer perceptron (MLP) to enable data transfer between different LIBS systems [32]. Transfer learning enables the knowledge established in the source domain to be transferred to target domains with limited samples or domain discrepancies, thereby effectively enhancing the efficiency of machine learning models. Despite these advantages, transfer learning also has limitations. It aligns features by modifying representations or model parameters without altering the original spectra. Its performance depends on sample size, architecture, and labels [33]. When there are large inherent discrepancies (e.g., differences in resolution or scale), it may fail to learn robust shared features. However, in real-world applications, differences in testing scenarios and instruments often lead to discrepancies in data sources, resulting in poor model generalization and limiting practical usability. Therefore, enabling the use of cross-instrument data within the model is of great significance for the real-world application of LIBS in TCM.

In this study, the data of three *Akebia* species were collected using two LIBS experimental systems (with spectrometers of different resolutions). The data collected from the low-resolution spectrometers were used to build the model, while the data from the high-resolution spectrometers were used for testing. Based on this framework, we propose a spectral correction and feature selection (SCFS) method to improve the generalization of the model. Furthermore, a post-processing (PP) strategy was applied to the test set to further enhance model performance.

## 2. Materials and Methods

### 2.1. Experiment Setup and Measurement

In this study, two LIBS experimental systems were utilized. The L-RLIBS setup consists of a low-resolution (L-R) spectrometer, whereas the H-RLIBS setup contains a high-resolution (H-R) spectrometer.

#### 2.1.1. L-RLIBS

**Figure 1:**
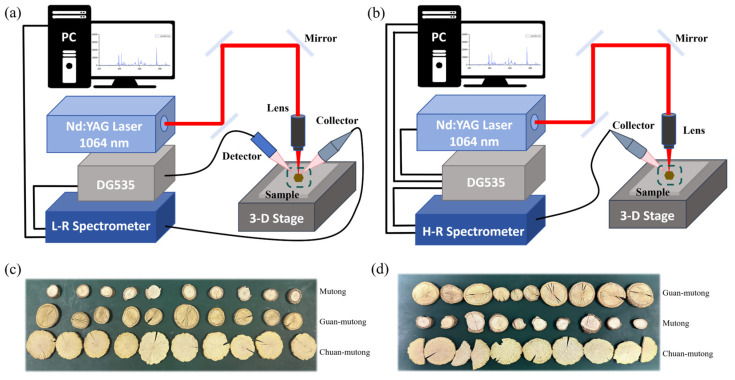
(**a**) Schematic of the L-RLIBS experimental setup. (**b**) Schematic of the H-RLIBS experimental setup. (**c**,**d**) *Akebia* species samples.

The L-RLIBS experimental setup is shown in Figure 1a. A homemade Q-switched Nd:YAG laser (wavelength 1064 nm, pulse energy 50 mJ, pulse width 10 ns) was employed to excite samples and generate plasma. The laser beam was reflected by three plane mirrors, passed through a lens (×10, working distance of 30.5 mm, M Plan Apo NIR), and focused onto the samples. Plasma emissions induced by the laser were collected by an optical collector and guided through an optical fiber into the spectrometer. A L-R spectrometer (AvaSpec 2048–2-USB2, Avantes, Apeldoorn, The Netherlands) was used for spectral data collection. This spectrometer is a dual-channel fiber optic spectrometer equipped with a charge coupled device (CCD), covering a spectral range of 190–1100 nm with a resolution of 0.2–0.3 nm. A photodetector detects the laser pulse and subsequently triggers a digital delay generator (SRS-DG535, Stanford Research Systems, Palo Alto, CA, USA), which in turn triggers the spectrometer for LIBS signal acquisition under software control. The laser repetition rate was 1 Hz, the delay time was set to 1.28 μs, and the CCD gate width was 1.05 ms.

All LIBS measurements were conducted under atmospheric conditions. Prior to measurements, the wavelength calibration of the spectrometers was performed using a Hg-Ar lamp. To minimize the influence of the thickness of each sample, the position and height of the 3D stage were adjusted before each measurement to ensure the laser is precisely focusing on the sample, thereby maximizing signal intensity. During measurement, the 3D stage was controlled by the software to move in such a way so that each laser pulse was directed at a fresh position on the sample. Each laser pulse induced a plasma emission that generated a single spectrum.

#### 2.1.2. H-RLIBS

The H-RLIBS experimental setup is shown in Figure 1b. The ablation source was a Q-switched Nd:YAG laser (Dawa−200, Beamtech Optronics, Beijing, China) operating at 1064 nm, a pulse energy of 50 mJ, and a pulse width of 10 ns. A H-R spectrometer (Aryelle 200, LTB Lasertechnik Berlin, Berlin, Germany) was used for spectral data collection. It is an echelle grating spectrometer equipped with an intensified charge coupled device (ICCD), covering 200–850 nm with a resolution of 0.01–0.02 nm. For the H-RLIBS, the DG535 serves as the central trigger source, simultaneously controlling the initiation of both the laser and the spectrometer, the laser repetition rate was 5 Hz, the delay time was set to 1 μs, and the ICCD gate width was 500 μs. Each spectrum was obtained by accumulating plasma emissions from 10 laser pulses.

### 2.2. Akebia Species Samples

The TCM samples used in this study included 20 slices each of Mutong, Chuan-mutong, and Guan-mutong. The data collected by the two systems were obtained from two separate batches of samples. As shown in Figure 1c,d, 10 slices were selected from each type of *Akebia* species medicines (a total of 30 slices) for measurement. Mutong and Chuan-mutong slices were purchased from Sichuan Ruifengcheng Traditional Chinese Medicine Co., Ltd., Chengdu, China and Guan-mutong was purchased from Anhui Yongxi Medical Technology Co., Ltd., Hefei, China Based on visual observation, the slices of Mutong range from ∅1 to ∅2 cm, with a yellowish-brown surface, slight curvature, and evenly distributed small pores. The slices of Chuan-mutong range from ∅2.5 to ∅ 3.5 cm, exhibiting a lighter color, hard texture, and irregular fractures along the edges. The slices of Guan-mutong range from ∅1 to ∅3 cm and have a complex surface texture and a yellow-brown coloration.

To simulate real-world scenarios of TCM circulation and identification, no pretreatment was performed on the samples prior to spectral acquisition. Then, the capability of LIBS to directly identify *Akebia* species medicines without any sample preparation could be evaluated and verified.

### 2.3. Data Analysis

#### 2.3.1. Data Pre-Processing

For each sample, 200 spectra were collected using the L-RLIBS, totaling 200 × 10 × 3 = 6000 spectra. Four spectra were averaged each time, reducing the dataset to 50 × 10 × 3 = 1500. In the H-RLIBS case, 50 spectra were collected per sample, and each spectrum was accumulated over 10 laser pulses; further averaging was unnecessary.

The L-RLIBS spectra were first processed using the Savitzky–Golay [34] algorithm to reduce noise. The principle of Savitzky–Golay filtering is to fit the original data within a moving window using a low-degree polynomial and then replace the central point with the fitted value, thereby achieving a smoothing effect. This preprocessing step was not applied to the H-RLIBS spectra. Finally, Max–Min normalization [35] was independently performed on the datasets from both systems.

(1) $$y=\frac{x-\min\left(x\right)}{\max\left(x\right)-\min\left(x\right)}$$

where $\max\left(x\right)$ is the largest intensity of the spectrum, and $\min\left(x\right)$ denotes the minimum intensity at the same wavelength.

#### 2.3.2. Feature Selection

This study employed the Analysis of Variance (ANOVA) method to quantitatively evaluate and rank the importance of spectral variables, retaining those with high discriminative power for subsequent modeling. ANOVA is a statistical method that tests the null hypothesis for each feature, which states that the feature has no average difference among different classes. The feature is considered of low importance if the *F*-test cannot reject the null hypothesis [36,37]. The *F*-value is calculated using the following formula.

(2) $$F=\frac{MSB}{MSW}=\frac{SSB}{k-1}\cdot \frac{N-k}{SSW}$$

where MSB is the mean square between classes, MSW is the mean square within classes, *SSB* is sum of squares between classes, *SSW* is the sum of squares within classes, *N* is the total number of samples, and k is the number of classes.

(3) $$SSB=\sum_{i=1}^{k}n_{i}{\left({\bar{x}}_{i}-\bar{x}\right)}^{2}$$

(4) $$SSW=\sum_{i=1}^{k}\sum_{j=1}^{n_{i}}{\left(x_{ij}-{\bar{x}}_{i}\right)}^{2}$$

where $n_{i}$ is the number of samples in the *i*th classes, ${\bar{x}}_{i}$ is the mean value of the *i*th classes, $\bar{x}$ is the overall mean of all samples, and $x_{ij}$ is the value of the *j*th sample in the *i*th classes.

By calculating the *F*-values, the importance of each feature can be assessed; a larger *F*-value indicates that the feature is more important.

### 2.4. Random Forest (RF) Algorithm

The classification model in this study is based on the random forest (RF) [38] algorithm, an ensemble learning method that performs classification or regression by aggregating the outputs of multiple decision trees. The core idea involves repeatedly sampling the training data with replacement (bootstrap sampling) and, during the construction of each tree, randomly selecting a subset of features for splitting to introduce model diversity. The optimal split at each node is determined using the Gini index [39]

(5) $$G=\sum_{k=1}^{k}p_{k}\left(1-p_{k}\right)$$

where k is the number of classes, and $p_{k}$ represents the probability that the spectra at a given node belong to a class.

Predictions for classification are made by majority voting. By combining multiple weak learners, RF exhibits strong resistance to overfitting and robust performance, making it a widely used technique for analyzing complex, high-dimensional, and small-sample datasets.

## 3. Results and Discussions

### 3.1. Spectra of Akebia Species Samples

**Figure 2:**
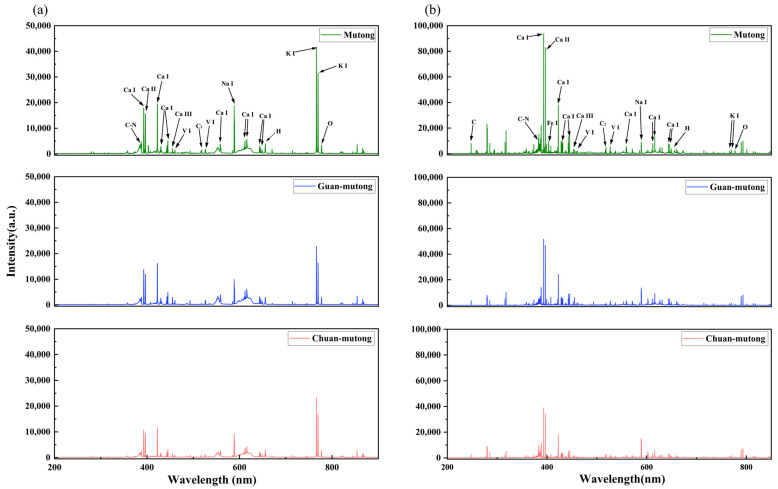
Averaged LIBS spectra acquired in the experiment. (**a**) L-RLIBS spectra. (**b**) H-RLIBS spectra.

Figure 2a,b show the averaged LIBS spectra of the three *Akebia* species acquired by the two systems. For the L-R fiber optic spectrometer, adjacent narrow spectral lines cannot be resolved, leading to fewer observable peaks and broader merged features. The H-R echelle spectrometer is capable of revealing complex peak structures, and it has a wider dynamic range of intensity, enabling the detection of weak signals. The L-R spectrometer is equipped with a CCD detector, which exhibits higher sensitivity in the infrared region than in the visible or ultraviolet regions, while the H-R spectrometer uses a UV-enhanced ICCD, resulting in higher responsivity in the ultraviolet region than in other spectral regions. However, these differences reflect the properties of the instruments rather than those of the samples.

The analysis and calibration of the LIBS spectra in Figure 2 are based on the atomic spectral database of the National Institute of Standards and Technology (NIST). The detected spectra reveal that the three *Akebia* species measured by the two LIBS systems have similar elemental emission lines, primarily including C, N, Ca, Fe, V, C_2_, Na, K, O, and H. These elements are mostly common components of organic groups or inorganic ions found in plants. Specifically, C, H, and O primarily originate from organic matrices, N is associated with nitrogen-containing compounds such as proteins and alkaloids, while Ca, K, Fe, V, and Na are likely related to mineral accumulation and the ecological growth conditions of the plants. Although the elemental composition is relatively consistent among the three *Akebia* species, differences in their geographic origins and active constituents lead to variations in the intensity of certain elemental spectral lines.

Due to the relatively fewer peaks in the L-RLIBS spectra and the absence of some peaks present in the H-RLIBS spectra, feature selection in this study was based solely on the L-RLIBS spectra, focusing on peak features with intensities greater than 1500. The selected peak features are those commonly detected by both systems, while for the same peak position, the measurements from the two systems exhibit certain fluctuations, with a shift ranging from 0.05 to 0.3 nm. Although O and H are commonly present in the samples, their spectral lines are prone to interference from atmospheric components such as O_2_ and H_2_O; therefore, O and H were excluded from the selected peak features to minimize background effects. In summary, a total of 20 peak features were selected through comparison and calibration with the NIST database, as listed in Table 1.

### 3.2. Model Building

#### 3.2.1. Classification Using Single-Instrument Data

RF models were separately built using the 20 selected peak features from the two systems, with the dataset split into training and test sets at a ratio of 7:3. For each sample class, the first 350 spectra were selected as the training set, while the subsequent 150 spectra were used for testing. The optimal parameters of RF are listed in Table 2.

**Figure 3:**
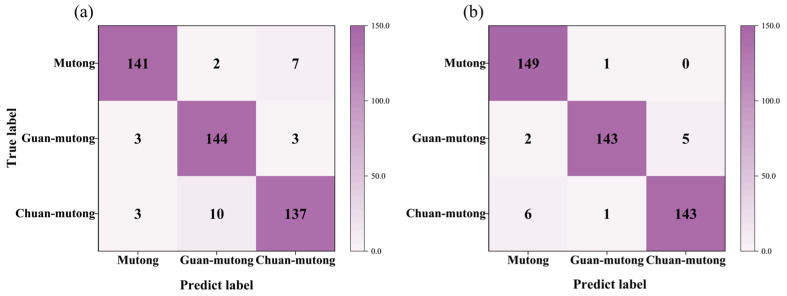
The confusion matrices based on the spectra with 20 features from (**a**) L-RLIBS and (**b**) H-RLIBS.

Figure 3a,b present the classification confusion matrices based on the 20 spectral features from the L-RLIBS and H-RLIBS, respectively. The classification accuracy reached 93.78% for the L-RLIBS data and 96.67% for the H-RLIBS data. Compared with the L-RLIBS spectra, the H-RLIBS spectra, even without preprocessing, achieved relatively higher classification accuracy. This is attributed to the higher sensitivity, dynamic range, and SNR of H-R spectrometer, which enhance the overall quality of the input data.

#### 3.2.2. Classification Using Cross-Instrument Data

**Figure 4:**
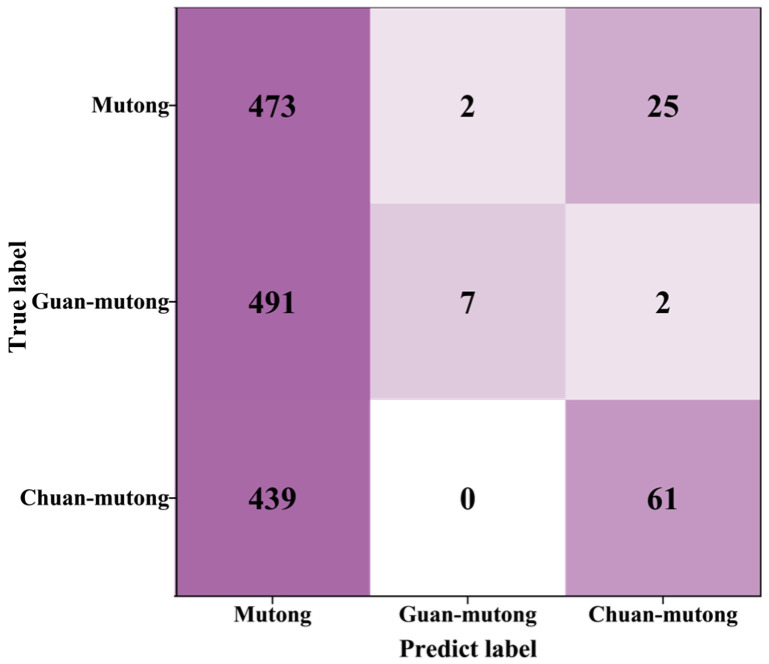
The confusion matrix for cross-instrument data utilization; spectra from L-RLIBS were used for training, while spectra from H-RLIBS were used for testing.

A total of 500 spectra with 20 features measured by L-RLIBS were used as the training set for RF, while 500 spectra with the same 20 features measured by H-RLIBS were used as the test set. Under this setting, the classification accuracy of the model on the H-RLIBS data was only 36.04%. Due to differences in spectral resolution, sensitivity, optical response, and noise between the two LIBS systems, the same features exhibit inconsistencies in peak shape details and intensity response. Although the peak wavelengths are consistent, differences between the two systems result in significant variations in peak shapes, which interfere with the model recognition of cross-instrument data and lead to poor classification performance. As shown in Figure 4, the model achieved relatively high accuracy in identifying Mutong, with a classification accuracy of 94.6%. However, most Guan-mutong and Chuan-mutong samples were misclassified as Mutong. This indicates that the model failed to effectively distinguish between the three species, likely due to the model learning only the features from the training set, while significant spectral differences exist between the training and test datasets.

### 3.3. Spectral Correction Combined with Feature Selection (SCFS)

#### 3.3.1. Spectral Correction Based on a Standard Lamp

**Figure 5:**
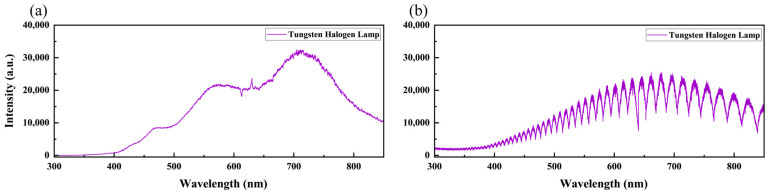
The standard lamp spectrum measured by (**a**) L-RLIBS and (**b**) H-RLIBS.

We adopted the standard lamp correction method in this study, a physically interpretable calibration approach that does not rely on sample size, architecture, and labels. Figure 5 shows the standard lamp spectra measured using the two LIBS systems. The standard lamp spectrum measured by the L-RLIBS is relatively smooth, as shown in Figure 5a. Figure 5b shows the spectrum measured by the H-RLIBS. For echelle spectrometers, higher-order diffraction can cause order overlapping, resulting in periodic peak-valley structures on the detector that resemble interference fringes.

The standard lamp provides a certified spectral range from 350 to 1000 nm, while the H-R and L-R spectrometers have detection ranges of 200–850 nm and 190–1100 nm, respectively. Therefore, the correction was limited to the overlapping range of 350–850 nm. By taking the ratio between the reference spectrum and the measured spectrum of the standard lamp, the wavelength-dependent response functions $R_{L}\left(\lambda \right)$ for L-RLIBS and $R_{H}\left(\lambda \right)$ for H-RLIBS can be obtained, respectively. These response functions can then be applied to adjust the spectral data. Since the spectral dimensions acquired by the two LIBS systems are different, we employ linear interpolation [40] to align the wavelength points across the datasets. The mathematical objective of the interpolation is to construct a continuous function $f\left(\lambda \right)$ that satisfies

(6) $$f\left(\lambda_{i}^{std}\right)=I_{std}\left(\lambda_{i}^{std}\right)$$

(7) $$I_{std}\left(\lambda^{new}\right)=f\left(\lambda^{new}\right)$$

where $\lambda_{i}^{std}$ is the known wavelength point within the standard lamp spectral range, and $\lambda^{new}$ is any wavelength point within that range.

**Figure 6:**
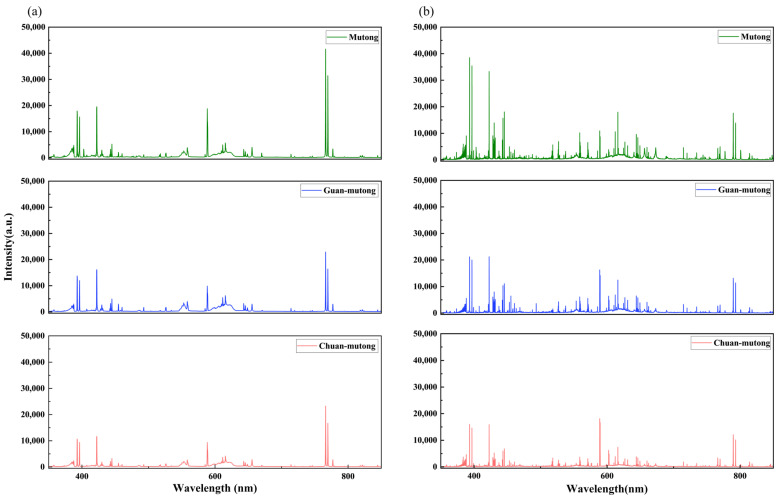
Comparison of (**a**) original L-RLIBS spectra and (**b**) corrected H-RLIBS spectra.

By taking the ratio between $R_{L}\left(\lambda \right)$ and $R_{H}\left(\lambda \right)$, a new response function $R_{L/H}\left(\lambda \right)$ for H-RLIBS can be obtained. This response function was used to correct the H-RLIBS spectra, and a comparison between the corrected H-RLIBS spectra and the original L-RLIBS spectra is shown in Figure 6. It is evident that after spectral correction, the discrepancies between the spectra are reduced, with improved similarity in peak, shapes, trends, and intensity ratios across the UV, visible, and infrared regions.

The corrected spectra with 20 features from the H-RLIBS were used as the test set and input into the previously established model. After spectral correction, the model achieved a classification accuracy of 69.49% on the test set.

#### 3.3.2. Feature Selection Based on ANOVA

**Figure 7:**
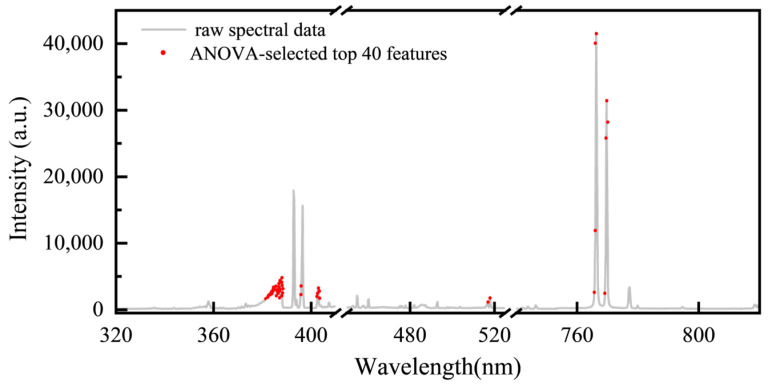
Top 40 ANOVA-selected spectral distributions.

To further improve the identification accuracy, we performed ANOVA on the wavelength points of the L-RLIBS data with intensity values greater than 1500. By calculating the *F*-values, an importance ranking of these wavelength points was obtained. The top 40 most discriminative wavelengths were mainly concentrated in the regions of 381–388 nm, 402–403 nm, and 766–769 nm, as shown in Figure 7.

*Akebia* species contain abundant alkaloids, proteins, and other organic compounds. So, the variation in organic composition among different *Akebia* species medicines leads to significant differences in the intensity of the CN band (381–388 nm). Both the 402–403 nm and 766–769 nm spectral regions correspond to the emission lines of metal elements derived from the soil. The 402–403 nm region contains weak emission lines for metals such as Fe, Ca, and Mn [41], whose accumulation in plants is influenced by regional differences in soil composition. The 766–769 nm region features two strong emission lines of K, another soil-derived metal that not only accumulates in plants but also plays a vital role in regulating cellular osmotic pressure and enzyme activation.

**Figure 8:**
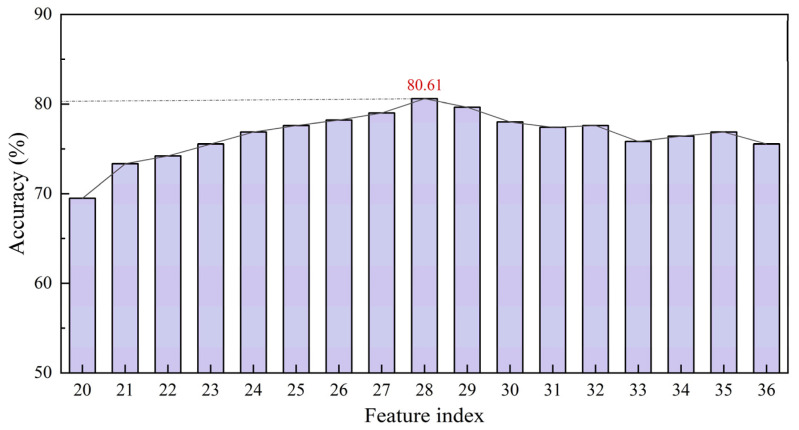
Classification accuracy of feature selection.

After feature selection evaluation on the L-RLIBS data, the same features were extracted from the H-RLIBS data by point-to-point matching, with the correspondence of characteristic wavelengths ensured through calibration against elemental emission lines from the NIST database. Starting from the initially selected 20 features, additional features were sequentially added to both training and test sets according to their *F*-values in descending order to assess the impact of feature quantity on classification accuracy after spectral correction. The experimental results indicate that the highest classification accuracy of cross-instrument data utilization was achieved when 28 features were included, as shown in Figure 8. As listed in Table 3, two of the features overlapped with the 20 peak-based features and were thus excluded.

**Figure 9:**
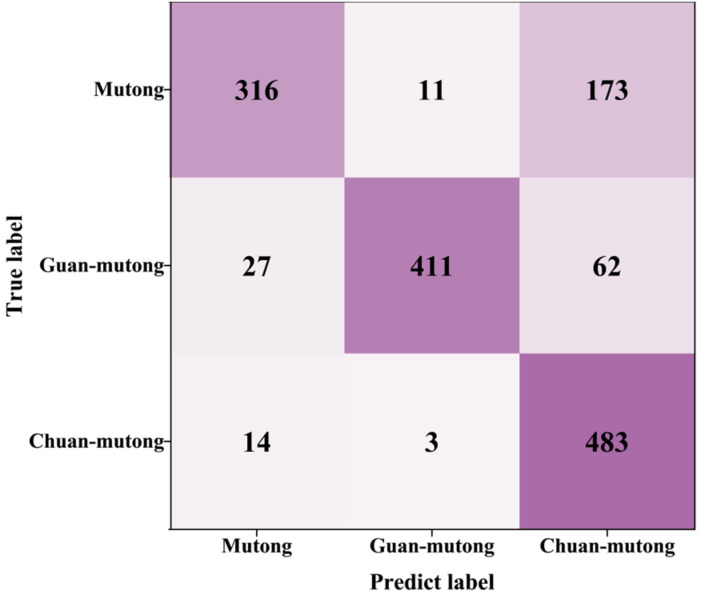
Confusion matrix after SCFS.

The confusion matrix after SCFS is shown in Figure 9. The classification accuracy of Mutong, Guan-mutong, and Chuan-mutong reached 80.61%. To evaluate the classification performance of the model on cross-instrument data utilization, the precision and recall for each class were calculated, as shown in Table 4.

The model demonstrated strong discriminative ability for Guan-mutong, achieving a high precision of 96.7% and a recall of 82.2%. Chuan-mutong exhibited the highest recall (96.6%); however, its precision was relatively low due to a considerable number of Mutong and Guan-mutong samples being misclassified as Chuan-mutong. In contrast, Mutong had the lowest recall, at only 63.2%, with many of its samples incorrectly predicted as Chuan-mutong. This misclassification may result from the high spectral similarity between Mutong and Chuan-mutong, making it difficult for the model to distinguish between Mutong and Chuan-mutong.

### 3.4. Post-Processing (PP)

In real-world applications, once the model has been trained, test data are typically fed into the model in a streaming manner. However, some of these data may be affected by measurement deviations and are therefore unsuitable as validation samples. Such data should instead be assigned to other categories or regarded as unrecognizable. Therefore, in order to further enhance prediction stability and to prevent overfitting during continuous model iteration and updating, a post-processing strategy was introduced as follows: unsupervised outlier removal from the test set using Density-Based Spatial Clustering of Applications with Noise (DBSCAN) [42,43] clustering. DBSCAN does not require a predefined number of clusters; it automatically identifies cluster structures by analyzing the density distribution of data points within the feature space. The principle of the algorithm is as follows: for a given data point, if the number of points within its specified radius (*ε*) reaches or exceeds a predefined minimum number of points (Min points), the point is considered “density-reachable” and assigned to a cluster; otherwise, the point is considered a border point or noise (an outlier).

**Figure 10:**
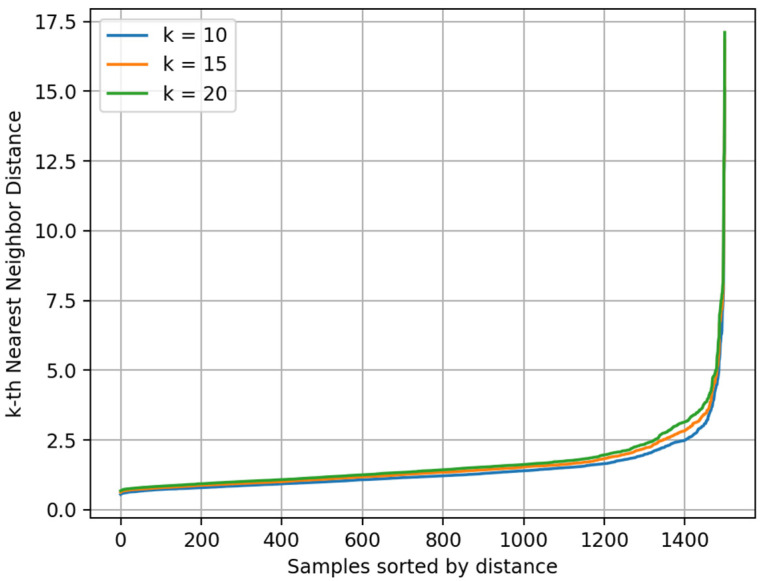
K-Distance Graph of test set.

To determine the optimal parameters for DBSCAN, we used the K-Distance Graph [44] method. This approach calculates the Euclidean distance between each sample and its *k*-th nearest neighbor, and it plots the distances in ascending order. In the resulting K-Distance Graph, as the sample index increases, the distance gradually increases. When the local density begins to drop, the curve typically shows a noticeable “elbow”. The corresponding distance at this elbow is considered a suitable ε value for clustering. Figure 10 presents the K-Distance Graph of the test set spectra for different *k* values (*k* = 10, 15, 20). Based on comparative analysis and fine-tuning, *ε* = 2.5 and Min points = 15 were ultimately selected as the optimal parameters for DBSCAN.

**Figure 11:**
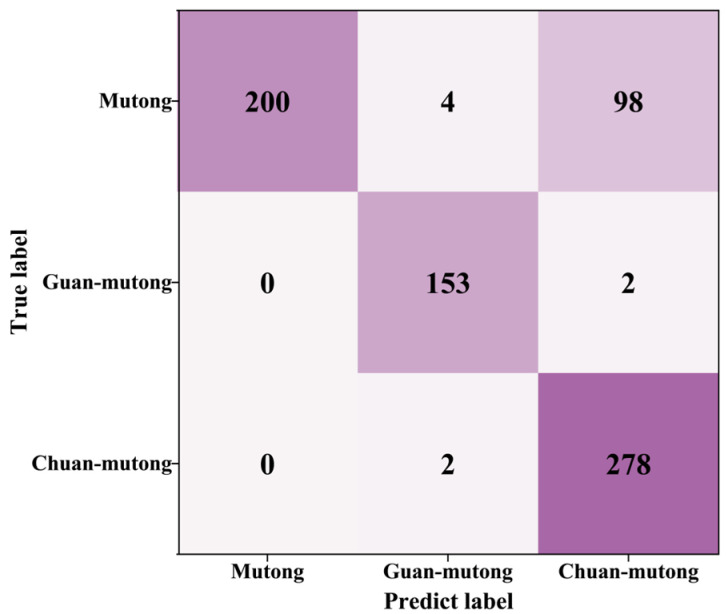
Confusion matrix after SCFS-PP.

After applying DBSCAN, 737 spectra remained in the test set, including 302 from Mutong, 155 from Guan-mutong, and 280 from Chuan-mutong. The confusion matrix after SCFS-PP is shown in Figure 11. The precision and recall for each class were calculated, as shown in Table 5.

**Figure 12:**
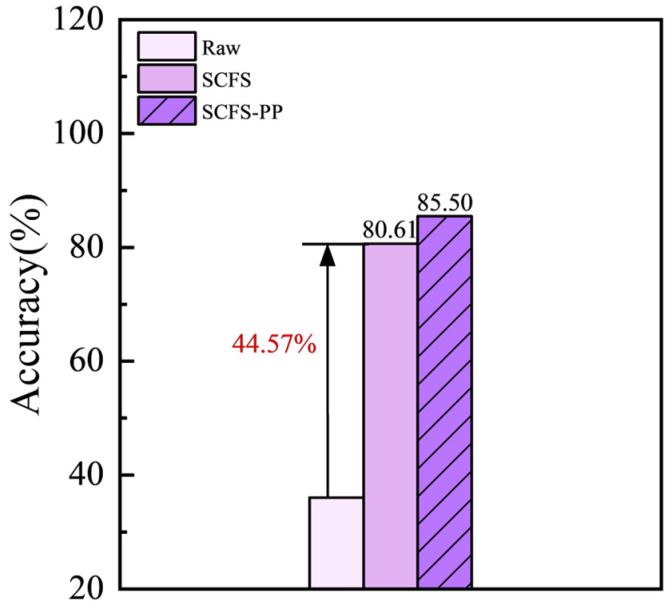
SCFS-PP method, improved classification accuracy of cross-instrument data utilization.

As shown in Figure 12 and Table 5, compared to the initial evaluation on the test set, the application of the PP strategy led to an improved classification accuracy of 85.50%, along with enhanced precision and recall for all classes. The model demonstrated strong performance in identifying Guan-mutong and Chuan-mutong. However, there was still significant confusion between Mutong and Chuan-mutong, with approximately 33% of Mutong samples misclassified as Chuan-mutong.

## 4. Conclusions

In this study, the LIBS spectra of three *Akebia* species were collected using two systems with spectrometers of different resolutions. For the data from L-RLIBS, Savitzky–Golay smoothing was applied as the preprocessing. RF models built using single-instrument data from L-RLIBS and H-RLIBS achieved classification accuracies of 93.78% and 96.67%, respectively. However, when using cross-instrument data, the classification accuracy of the model dropped to only 36.04%. To address this issue, we proposed the SCFS method. In this approach, spectral data were first corrected using a standard lamp, followed by feature selection based on ANOVA to identify the most discriminative features. The highest classification accuracy of 80.61% was achieved when 28 features were used. To further improve model performance, a PP strategy was applied where abnormal spectra in the test set were removed using DBSCAN, resulting in a final classification accuracy of 85.50%. The experimental results demonstrate the effectiveness of the “SCFS-PP” framework in improving the generalization of model for cross-instrument data utilization. This approach offers technical support for the quality control of TCM products.

## Figures and Tables

**Table 1:** Elements and molecular fragments corresponding to the 20 selected peak features.

Elements and Molecular Fragments	Wavelength (nm)
C-N	383.84, 387.68
Ca I	392.35, 422.67, 429.89, 445.48, 558.19, 612.22, 616.22, 643.91, 646.26
Ca II	396.85
Ca III	455.33
Fe I	402.96
V I	460.61, 526.61
C_2_	517.90
Na I	588.99
K I	766.49, 769.90

**Table 2:** The optimal parameters.

Model	Optimal Parameters	Values
RF	The number of decision trees	500
	Minimum leaf size	20

**Table 3:** Top 10 most important features selected by the ANOVA method.

No.	Wavelength (nm)	*F*-Value	Status
1	402.69	825.280	Newly added
2	402.96	750.798	Already included
3	383.84	533.167	Already included
4	382.86	522.757	Newly added
5	402.41	522.525	Newly added
6	383.14	514.171	Newly added
7	382.00	514.151	Newly added
8	382.57	488.141	Newly added
9	381.72	483.645	Newly added
10	395.90	464.441	Newly added

**Table 4:** Precision and recall for each class after SCFS.

Class	Precision (%)	Recall (%)
Mutong	88.55	63.2
Guan-mutong	96.7	82.2
Chuan-mutong	67.3	96.6

**Table 5:** Precision and recall for each class after SCFS-PP.

Class	Precision (%)	Recall (%)
Mutong	100	66.2
Guan-mutong	95.6	98.7
Chuan-mutong	73.9	99.3

## Data Availability

Data is contained within the article.
